# Integrated multiomics analysis to infer COVID-19 biological insights

**DOI:** 10.1038/s41598-023-28816-5

**Published:** 2023-01-31

**Authors:** Mahmoud Sameh, Hossam M. Khalaf, Ali Mostafa Anwar, Aya Osama, Eman Ali Ahmed, Sebaey Mahgoub, Shahd Ezzeldin, Anthony Tanios, Mostafa Alfishawy, Azza Farag Said, Maged Salah Mohamed, Ahmed A. Sayed, Sameh Magdeldin

**Affiliations:** 1 Basic Research Department, Proteomics and Metabolomics Research Program, Children’s Cancer Hospital 57357 (CCHE-57357), Cairo, Egypt; 2 Intensive Care Unit, As-Salam International Hospital, Cairo, Egypt; 3 Department of Pharmacology, Faculty of Veterinary Medicine, Suez Canal University, Ismailia, 41522 Egypt; 4 Infectious Diseases Consultants and Academic Researchers of Egypt (IDCARE), Cairo, Egypt; 5 Alazhar Center for Allergy and Immunology, Cairo, Egypt; 6 Department of Pulmonary Medicine, Faculty of Medicine, Minia University, Minia, Egypt; 7 Department of Anesthesia and Intensive Care, Kasr Al Ainy, Cairo University, Cairo, Egypt; 8 Department of Basic Research, Genomics Program, Children’s Cancer Hospital 57357, Cairo, Egypt; 9 Department of Biochemistry, Faculty of Science, Ain Shams University, Cairo, Egypt; 10 Department of Physiology, Faculty of Veterinary Medicine, Suez Canal University, Ismailia, Egypt

**Keywords:** Systems biology, Metabolomics, Proteins, Proteomics

## Abstract

Three years after the pandemic, we still have an imprecise comprehension of the pathogen landscape and we are left with an urgent need for early detection methods and effective therapy for severe COVID-19 patients. The implications of infection go beyond pulmonary damage since the virus hijacks the host's cellular machinery and consumes its resources. Here, we profiled the plasma proteome and metabolome of a cohort of 57 control and severe COVID-19 cases using high-resolution mass spectrometry. We analyzed their proteome and metabolome profiles with multiple depths and methodologies as conventional single omics analysis and other multi-omics integrative methods to obtain the most comprehensive method that portrays an in-depth molecular landscape of the disease. Our findings revealed that integrating the knowledge-based and statistical-based techniques (knowledge-statistical network) outperformed other methods not only on the pathway detection level but even on the number of features detected within pathways. The versatile usage of this approach could provide us with a better understanding of the molecular mechanisms behind any biological system and provide multi-dimensional therapeutic solutions by simultaneously targeting more than one pathogenic factor.

## Introduction

On December 31, 2019, a cluster of “Viral pneumonia” cases from an unknown source was reported in Wuhan, China, with a nearly consistent plethora of symptoms similar to severe acute respiratory syndrome (SARS). Symptoms including fever, persistent dry cough, shortness of breath, loss of taste or smell, and headache were commonly noticed and varied according to the clinical status of patients. Cases were graded into; mild, moderate, severe, and critical based on the level of cellular damage severity in the lungs, ranging from asymptomatic to life-threatening^1^. Far from the typical epidemiological and clinical characteristics of most COVID-19 patients caused by the immune response, they also show a lot of dysregulated biological processes like coagulation, degranulation of immune components, lipid, fatty acids, and amino acids metabolism^2^. The novel beta-coronavirus pathogen has been identified as a highly transmissible single-stranded RNA virus strain of the *Coronaviridae* family^3^. First, it was named the new coronavirus 2019 (nCOV-2019) until it was recently renamed a (SARS-CoV 2) by the international committee on taxonomy of viruses due to its high homology to the older (SARS-COV)^4,5^. Despite all of the rigorous preventive measures and the scientific community's efforts to find new ways for fast and accurate diagnostic tools and therapies for (COVID-19), the virus has rapidly spread all over the world, causing more than 6.4 million deaths as of September 2022, according to WHO COVID-19 dashboard^3^. The viral genomic structure shares up to 79.5% similarity to the previous SARS-COV, while only 50% similarity to the MERS-COV^6,7^. However, these differences in the genetic makeup reflect possible vital proteome changes that lead to much higher infectivity and transmissibility than any other (SARS) strain^8^.

The viral proteomic structure is composed of nonstructural proteins encoding enzymes and transcription factors indispensable for the virus replication that are only present in the infected host, and other four main structural proteins: S (spike), E (envelope), M (membrane), and N (nucleocapsid). These proteins assimilate the virion particle of (SARS-COV 2) with different functions^9^. Mechanistically, the S protein is responsible for the viral cell entry by binding to the human angiotensin-converting enzyme 2 (ACE2) via the S1 and S2 subunits on the surface of the spike glycoprotein, where the receptor-binding domain (RBD) of the S1 subunit attaches to the (ACE2). In contrast, S2 subunit contributes to the membrane fusion and viral internalization process^6,9^. The expression of the ACE2 and TMPRSS2 is highly relative from one organism to another and not exclusive to the lung cells. Both receptors were coexpressed in the ileum and colon^10^. This observation suggests that other viral entry routes through the digestive system might be involved^10,11^. The viral cell entry process triggers an immune response on two levels; the innate immune response against damaged tissues leads to acute respiratory distress syndrome (ARDS), characterized by rapid lung inflammation and respiratory failure. A process relies heavily on the antiviral activity of the interferon (IFN) I. On the other hand, the adaptive immune response that develops post-infection or vaccination relies on producing specific immunoglobulins and T-cells with viral recognition and elimination properties^12,13^. The mystery of the pathogen etiology has been primordially deciphered without a clear understanding of the biological changes on the molecular level of biomolecules such as proteins, metabolites, and lipids. Here, we utilized a high-resolution mass spectrometry (MS) to screen and analyze the molecular plasma proteome and metabolome of COVID-19 patients using different methods, including single-omics and three different multi-omics approaches. Intriguingly, multi-omics approaches harnessed a preferential biological enrichment vision of the SARS-CoV 2. In addition, our study precisely describes the biological impact of the different omics analyzing methods.

## Materials and methods

### Blood collection and processing

A cohort of 32 patients with sever COVID-19 and 25 healthy control individuals were enrolled in a monocentric observational study conducted in the unit of proteomics and metabolomics at 57357 Children's Cancer Hospital, Egypt, according to the declaration of Helsinki, and under standard clinical practice guidelines. Only participants with informed consent were eligible to participate in the study. The research ethics committee at the ministry of health and population (IRB0000687) approved all experimental procedures. All contributors were either positively or negatively confirmed by SARS-CoV-2 nucleic acid RT-qPCR testing. Negative cases were free from influenza-like symptoms during sample collection and 2 weeks before.

Moreover, positive cases were further confirmed by radiological and clinical examination according to CDC guidelines^14^. Blood samples were collected from Misr International Hospital and treated according to the biocontainment procedures of the SARS-COV-2-samples^14^, where all of the blood samples were collected directly in 10 ml EDTA tubes and processed one day after the blood draw. Plasma was obtained after whole blood centrifugation at 1400 g for 10 min at 4 °C, then aliquoted into cryovials and preserved at −80 °C for further proteomics and metabolomics analysis.

### COVID-19 proteome analysis

#### Extraction of plasma proteins

For plasma proteins extraction, 8 M Urea lysis buffer (8 M urea, 500 mM Tris HCl, pH 8.5) with 1ul of stock solution of protease and phosphatases inhibitor cocktail (complete ultra-tablets, mini, Roche, Mannheim) were added for protein denaturation and inhibition of any possible protease activity. After incubation at 37 °C for 1 h with vigorous vortexing, samples were centrifuged at 12,000 rpm for 20 min. The lysate was then precipitated with chilled acetone at −20 °C. Samples were centrifuged at 10,000 rpm for 30 min at 4 °C. Extracted proteins were collected and assayed using bicinchoninic acid assay (BCA assay, Pierce, Rockford IL) at Å562 nm for quantification.

### Protein lysate tryptic digestion

Thirty μg protein extract was reduced and alkylated with 2 μl of 200 mM Dithiothreitol (DTT) and 2 μl of 1 M Iodoacetamide (IAA), respectively. Samples were then diluted with 100 mM Tris pH 8.5 to reach around 2 M urea before trypsinization. 1 μg/μl Trypsin was added for protein digestion at a ratio of 1:30 (enzyme: protein) (Sigma, Germany) and incubated overnight in a thermo-shaker at 600 rpm at 37 °C. Digested peptides were then desalted using C18 StageTips (Pierce™ C18 Spin Tips; prod#84850)^15^. Digested peptides solution was acidified using 90% formic acid to a final pH of 2.0, dried, and reconstituted in 0.1% formic acid.

### Nano-LC MS/MS analysis

Nano-LC MS/MS analysis was carried out on Eksigent nanoLC 400 autosampler with Ekspert nanoLC 425 pump coupled with TripleTOF 5600^+^ mass spectrometer (AB SCIEX, Concord, Canada). For peptide trapping, 500 ng of peptides (10 µl) were trapped on CHROMXP C18-CL 5um (10 × 0.5 mm) (SCIEX, Canada). The tryptic digest mixture was eluted by one step linear gradient of mobile phase solvent B (100% ACN, 0.0.1% formic acid) from 3 to 80% for 2 h of separation. The column temperature was held at 30 °C, and the flow rate was set to 5µL/min. MS and MS/MS scan range was set from 400 to 1250 m/z and 170–1500 m/z, respectively. Collision-induced dissociation (CID) was used as a source of fragmentation. The source voltage was set to 5500 V, and the curtain gas was 10 psi. The analytical column was 3 µm, ChromXP C18CL, 120 A, 150 × 0.3 mm (SCIEX, Canada). The most 40 intense ions were sequentially registered in data-dependent acquisition (DDA) positive mode with charge state 2–5. Calibration was performed by injecting PepcalMix (SCIEX, Canada) before and within the batches and scheduled to be injected every six hours to prevent mass shift and correct possible TOF deviation. Plasma samples were randomly distributed in every batch. The mass spectrometer performance was consistently evaluated with external calibration before analysis.

### Proteins identification and LFQ quantification

Mascot generic format (mgf) files were generated from raw files using a script supplied by AB Sciex. MS/MS spectra of two types of samples were searched using X Tandem in Peptide shaker (version 1.16.26). Samples were searched against UniProt *Homosapiens* (Swiss-Prot and TrEMBL database containing 176,507 proteins) with target and decoy sequences (downloaded on 8.3.2021)^16^. The search space included all fully and semi-tryptic peptide candidates with a maximum of 2 missed cleavages. Precursor and fragment mass were identified with an initial mass tolerance of 20 ppm and 10 ppm, respectively. Carbamidomethylation of cysteine (+ 57.02 amu) was considered as a static modification and oxidation at Methionine (+ 15.99 amu), Acetylation of protein N- terminal and K (+ 42.01 amu), and pyrrolidone from carbamidomethylated C (−17.03 amu) as variable modification. The false discovery rate (FDR) was kept at 1% at the protein level to ensure a high-quality result. Final assembly of sample replicates was applied to generate the final outputs of each sample using in-house software ‘ProteoSelector’ (https://www.57357.org/en/department /proteomics-unit-dept/in-house-bioinformatics-tools/). Samples were normalized using probabilistic quotient normalization (PQN)^17^. The latter transforms proteins/metabolites spectra according to an overall estimation on the most probable dilution.

### COVID-19 Metabolome analysis

#### Extraction of small molecules for metabolomics

Fifty µL of plasma aliquots were used for metabolites extraction. Briefly, each sample was treated with 300 μl of a precooled extraction solvent consisting of chloroform: water: methanol with a ratio of 1:1:3, respectively. The mixture was vortexed and whirled for at least 2 min, followed by ultrasonication for 15 min at 4 °C, and centrifuged at 12,000 rpm at 4 °C for 10 min. Samples were further dried using Eppendorf concentrator plus at 30 °C. The dried extract was reconstituted in a solvent consisting of water: methanol: acetonitrile with a ratio of 2:1:1, respectively, and used for UHPLC-MS/MS analysis.

### UHPLC-MS/MS analysis (SWATH acquisition method)

Extracted samples were subjected to LC–MS/MS analysis in both positive and negative ionization modes using SWATH™ acquisition as a data-independent acquisition (DIA) to detect small molecules with low background noise and to avoid the bias toward the most intense signals from the sample^18^. UHPLC-MS/MS analysis was performed using ExionLC™ AC UHPLC system (AB SCIEX, Concord, Canada) with Acquity XSelect HSS T3 analytical column 2.1 × 150 mm, 2.5 µm (Waters Co, Milford, US) coupled with TripleTOF 5600^+^ mass spectrometer (AB SCIEX, Concord, Canada). For chromatographic separation, 10 μl of the sample was eluted for 28 min using gradient elution with a steady flow rate of 0.3 ml/ min. The mobile phase solutions which consist of solution (A); 5 mM ammonium formate in 1% methanol (pH 3.0) for positive mode, solution (B); acetonitrile, and solution (C); 5 mM ammonium formate in 1% methanol (pH 8.0) for negative mode elution. Gradient elution was scheduled as follows: 0% B for 1.0 min, 0–90% B in 20 min, 90% for 4.0 min, 90–0% B in 1.0 min, and finally, re-equilibrating with 0% B for 3.0 min was sustained. Mass spectrometric analysis was performed using a DuoSpray™ ion source for positive-ion (ESI^+^) and negative-ion (ESI^–^) modes. Sequential windowed acquisition of all theoretical fragment ion MS (SWATH) method was used to detect metabolites within the samples by setting a single TOF scan from 50 to 1100 Da accumulated in 30 ms followed by product ion TOF scan from 50 to 1100 Da using a fixed 50 Da transition windows. The SWATH-acquired files were analyzed using SASA^19^. MS and MS/MS spectra were captured using Analyst TF (v 1.7.1). Quality control (QC) measures for metabolomics analysis were done by injecting QC samples pooled from 5 μl of each sample after every 10 samples in the running sequence to monitor the quality and stability of the run. Automatic Mass calibration was performed every 2 h using APCI calibration solution (AB SCIEX, Canada) (Fig. S1).

### Metabolites identification and quantification

PeakView 2.2, MasterView 1.1 package (AB SCIEX), and MultiQuant Software (AB SCIEX) were used to detect parents and fragment ions. Precursor ion XIC signal to noise ratio (S/N) > 3, sample: blank > 5 with a precursor mass tolerance of 10 ppm were applied as search parameters. High-resolution Human Metabolome database containing 114,100 metabolites (HMDB; downloaded March 2021) was used as a search space. For HMDB, fragments relative intensities per component (SPLASH) were filtered and stratified depending on their relative intensity. Samples were normalized using total ion chromatogram (TIC). Small molecules abundance data files were aggregated into one file using an in-house software tool and subjected to statistical analysis.

### Single omics data analysis

To ensure data quality, proteins and metabolites quantified in < 20% of samples/each group of the SARS-Cov2 cohort were excluded. In addition, drugs and their metabolites were manually removed from the metabolites profile before downstream analysis. Proteins and metabolites that passed the missing value criteria (< 20%) were subjected to a modified imputation method based on the conventional median imputation^20^, where missing values were imputed with random values ranging from +1 to −1% around the median value of each group. After imputation, samples were normalized using probabilistic quotient normalization (PQN). Auto-scaling (mean-centered and divided by the standard deviation of each variable) was performed on both proteomics and metabolomics datasets. Unpaired t-test with *p* value ≤ 0.05 and adjusted *p* value (FDR) ≤ 0.1 were applied. Additionally, a fold-change cut-off was employed to obtain the differentially expressed features. Both differentially expressed proteins (DEPs) and metabolites (DEMs) were subjected to hierarchical clustering analysis, volcano plot, and Principal Component Analysis (PCA) using MetaboAnalystR 5.0^21^. Gene ontology and pathway enrichment analysis were retrieved from KEGG and Reactome using g: profiler^22^. R project for statistical computing was used for graphical representation^23^.

### Multi-omics analysis

#### Knowledge-based multi-omics approach

Two hundred and sixty proteins and 44 metabolites that passed missing value criteria coupled with uniquely expressed proteins and metabolites in the COVID-19 cohort were used as seeds for constructing a knowledge-based network. The latter illustrates the highly confident interactions between plasma proteins and metabolites in COVID-19 patients extracted from STITCH^24^. The knowledge-based network was extracted and visualized using Cytoscape 3.9.0^25^. Moreover, we investigated the biological impact of the network entities through the KEGG-Reactome pathway enrichment analysis to demonstrate the biological pathways that were uncovered and enriched by the network.

### Statistical-based multi-omics approach

For key feature selection and correlation pattern detection across omics layers, we applied a data-driven approach for multi-omics integration using OmicsAnalyst to navigate the complex landscape of our proteome and metabolome profiles through intuitive visual analytics^26^. The same set of proteins and metabolites used for the knowledge-based analysis were recruited for the statistical-based analysis. The Normalized spectral abundance factor (NSAF) was scaled to match the metabolites' abundances. Afterward, both datasets passed the quality assessment. They were represented by density plot, PCA plot, and t-distributed stochastic neighbor embedding (t-SNE) plot to provide an overview of the distribution of the omics profiles. Alongside highlighting similarities and differences between the different samples using linear “PCA” and nonlinear “t-SNE” transformation. We constructed a correlation network based on a multivariate feature selection method called DIABLO (Data Integration Analysis for Biomarker discovery using Latent components) coupled with a Pearson similarity matrix with a maximum of 25 features per component to compute the pairwise similarity between selected features^27^. We also applied a dimension reduction analysis based on Multiple co-inertia analysis (MCIA) to visualize the sample loading on 2D and 3D spacing^28^. Finally, KEGG-Reactome pathway enrichment analysis was applied to investigate the enriched biological pathways by the network entities.

### Integrated knowledge-statistics-based approach

In this approach, both knowledge and statistical-based networks were merged. Shared proteins and metabolites found in both knowledge and statistical-based networks were selected with their first shell interactors to construct a new network with features integrated on the knowledge-based level and correlated on the statistical level. The new integrative network was constructed and visualized using Cytoscape 3.9.0. This new knowledge-statistical network was subjected to KEGG-Reactome pathway enrichment analysis to compare its biological impact to prior methods.

### Comparative network and pathways analysis

We compared all the resulting networks based on the number of seeds and edges for each network using the UpSet plot. In addition, we calculated the distance between each network according to the Jaccard similarity index to elucidate the similarities between the networks^29^. Furthermore, we compared the enriched pathways (FDR < 0.05) uncovered by each method and visualized them using venn diagram to illustrate the significant shared biological events between these methods and unique ones. To be more focused, we picked four biological pathways consistent with the SARS-CoV-2 pathophysiology, including Platelet activation, signaling and aggregation pathway, Coronavirus disease 2019 (COVID-19) pathway, Disorders of transmembrane transporters pathway, and Purine metabolism pathway. We tracked their molecular pathway changes in terms of the pathway's significance and the number of entities across the single and multi-omics approaches to clarify the profound biological impact of each method.

## Results

### Proteomic and metabolomic profiling of COVID-19 patients

**Figure 1:**
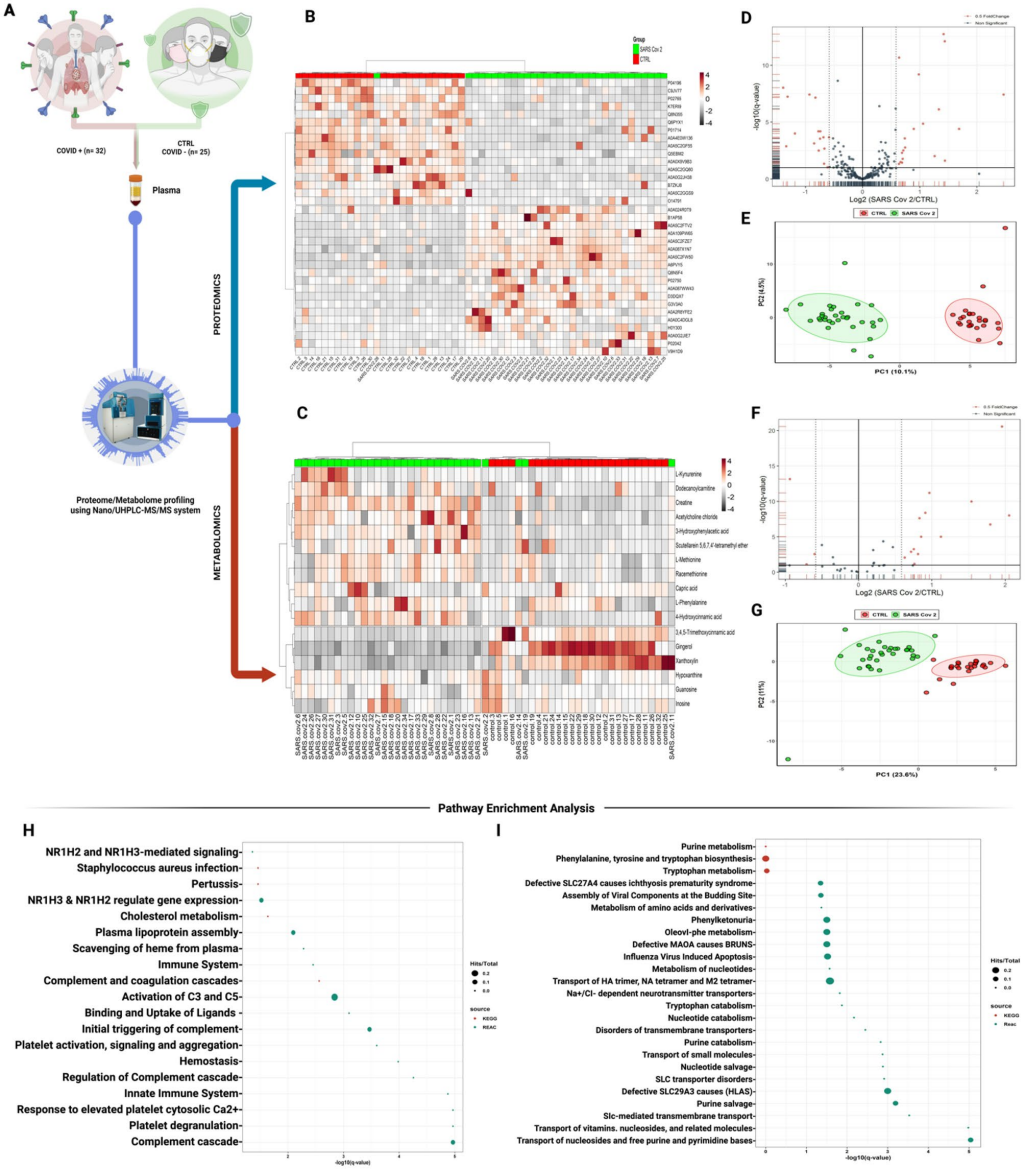
Proteomic and metabolomic analysis of plasma from COVID-19 patients. (**A**) Holistic view of the experimental design starts with plasma extraction from 57 individuals: 25 healthy individuals and 32 severely diagnosed SARS-CoV-2-positive patients. (**B**, **C**) Heat map hierarchical clustering highlights the top 25 significant proteins and metabolites between the two groups, respectively. (**D**, **F**) Volcano plot analysis showing DEPs and DEMs, respectively (**E**, **G**) Principle component analysis for proteomics and metabolomics profiles, respectively. (**H**, **I**) pathway enrichment analysis of untargeted proteomics (**H**) and metabolomics (**I**) retrieved from KEGG and Reactome databases using proteins and metabolites passed FDR 5% (q-value) and *p* value < 0.05. Colours represent different databases. The size of the circle reflects the identified number of hits over the total pathway entities. The x-axis represents the –log10 of the q-value.

A holistic view of the experimental design is illustrated in (Fig. 1A). Briefly, we examined a cohort of 32 COVID-19 patients with severe symptoms and 25 healthy individuals (negative for the SARS-CoV-2). We used an untargeted Nano/UHPLC-MS/MS approach to screen and analyze the two cohorts' plasma proteins and metabolites profiles. Coherently, 2585 proteins and 589 metabolites (including 58 drugs and their metabolites) were identified and relatively quantified. Despite excluding drugs from the following analysis, the study highlights our profiling approach's efficiency and reflects the samples' complexity and the patient's exposure to different therapies. Among identified macromolecules, 252 proteins and 42 metabolites passed the above-mentioned missing values criteria. Ultimately, 35 proteins and 17 metabolites passed the t-test with a *p* value cut-off ≤ 0.05 and adjusted *p* value (FDR) cut-off ≤ 0.1. This set of proteins and metabolites was subjected to univariate, chemometrics, and clustering analysis to illustrate the differentially expressed features in each profile. Most DEPs were acute-phase proteins, immunoglobulins, and conjugated proteins like apolipoproteins (Fig. S2). Most DEMs consisted of organic compounds, amino acids, and their derivatives (Fig. S3).

Heatmaps analysis showed the top 25 proteins and metabolites with feature and sample clustering (Fig. 1B,C). The volcano plot showed 35 DEPs passing the 0.5 fold change cut-off and 17 DEMs passing the 1.5 fold change cut-off (Fig. 1D,F, Table S1). Unambiguously, Principal component analysis (PCA) revealed a stratified pattern between SARS-CoV-2 and healthy controls plasma proteins and metabolites profiles, as shown in Fig. 1E,G. DEPs and DEMs were subjected to Gene Ontology (GO) and pathway enrichment analysis. As illustrated in (Fig. S4), the GO terms revealed biological processes related to complement activation, endocytosis, and acute-phase response dysregulation of endopeptidase activity and lipase inhibitor activity. The extracellular space and lipoproteins particles were among the most activated cellular components. KEGG-Reactome pathways were enriched in biological pathways mostly related to inflammatory response, platelet degranulation, complement and coagulation cascade, small molecules transport disorder, amino acids, purines metabolism, and catabolism dysregulation (Fig. 1H,I).

### Knowledge-based network analysis

**Figure 2:**
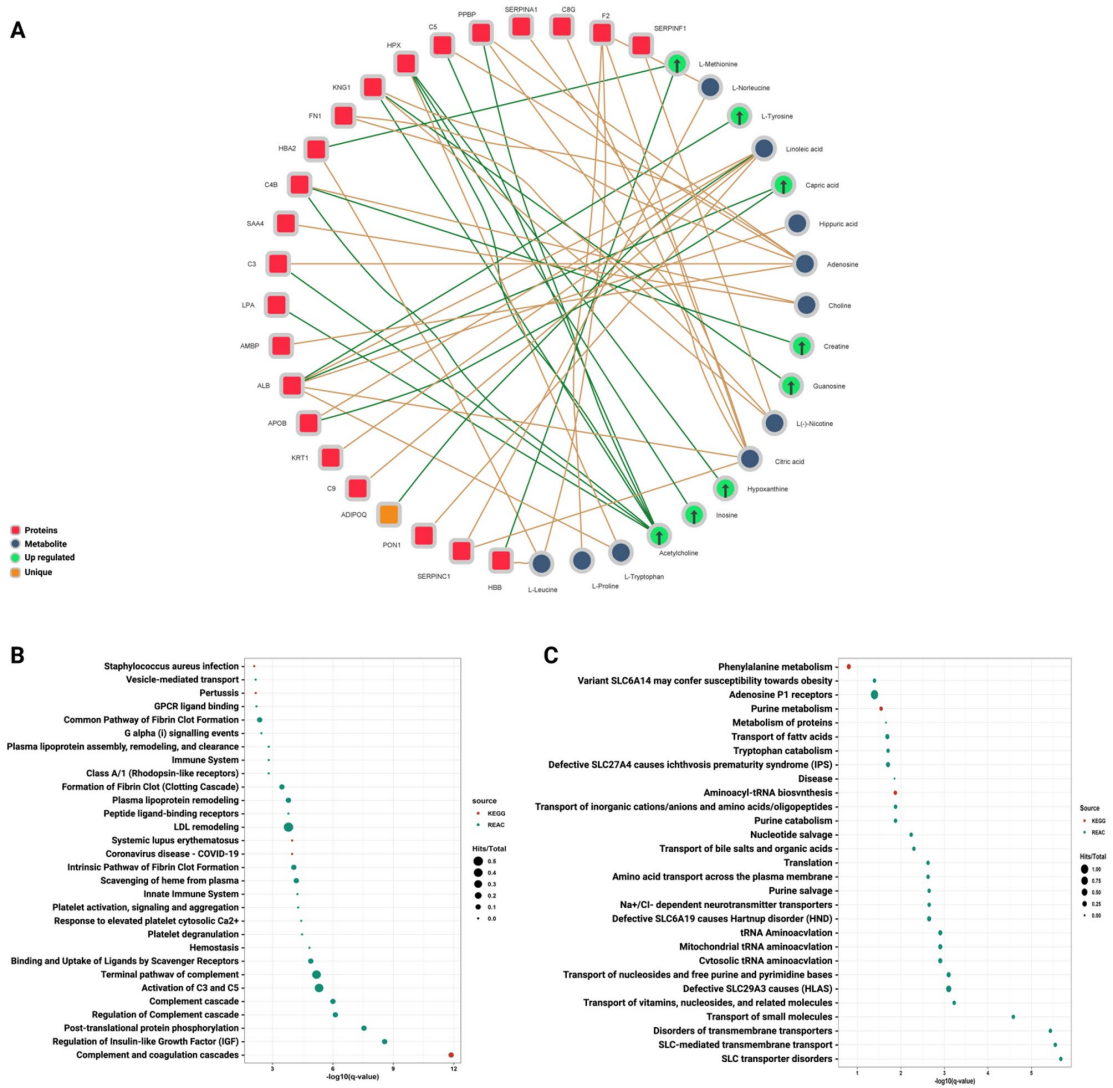
Knowledge-based interactions between plasma proteins and metabolites. The knowledge-based network generated by MetaboAnalyst and visualized by Cytoscape. (**A**) The network shows the metabolite-protein interactions across both omics The network contained 14 proteins, 18 metabolites, and 48 protein-metabolite interactions. Proteins are labelled in red and metabolites in blue. Upregulated and unique molecules are tagged in green and orange, respectively. (**B**, **C**) Pathway enrichment analyses applied on the knowledge-based network and retrieved from KEGG (red) and Reactome (green) databases. The circle size reflects the identified hits in this pathway divided by the total entities and the x-axis is the –log10 of the q-value.

We hypothesized that integration between biomolecules across different omics on the level of pre-existed knowledge would clarify the molecular landscape of the SARS-Cov2 infection and uncover new functional connections between plasma proteins and metabolites that have not been unleashed by single omics analysis. Therefore, we integrated 260 proteins with 44 metabolites representing features that only passed the missing value criteria and unique features in the COVID-19 cohort’s plasma. Certainly, we did not use the whole omics profiles in order not to fall into the “hairball” effect caused by the large network seeds and to focus on significant and unique features implicated in SARS-Cov2 infection. Only 23 proteins and 18 metabolites were found to have a highly confident gene-metabolite association and used to build one continent network consisting of 41 nodes and 48 edges (Fig. 2A). Interestingly, the network contained 8 upregulated metabolites: L-Methionine, L-Tyrosine, Hypoxanthine, Inosine, Creatine, Guanosine, Capric acid, Acetylcholine, and 1 unique protein: Adiponectin (Fig. 2A, Table S2). Pathway enrichment analysis applied to the network proteins and metabolites shared 71% of the single omics pathway signature with higher enrichment values in most pathways due to introducing new proteins and metabolites hits (Fig. 2B,C). Also, the knowledge-based interactions introduced new enriched pathways that were congruent with earlier studies on SARS-CoV-2 or even by the single omics approach, including coronavirus disease—COVID-19, terminal pathway of complement, formation of fibrin clot (Clotting Cascade), plasma lipoprotein remodelling and aminoacyl-tRNA biosynthesis. This observation is compatible with previous investigations on SARS-CoV-2^30,31^.

### Statistical-based network analysis

**Figure 3:**
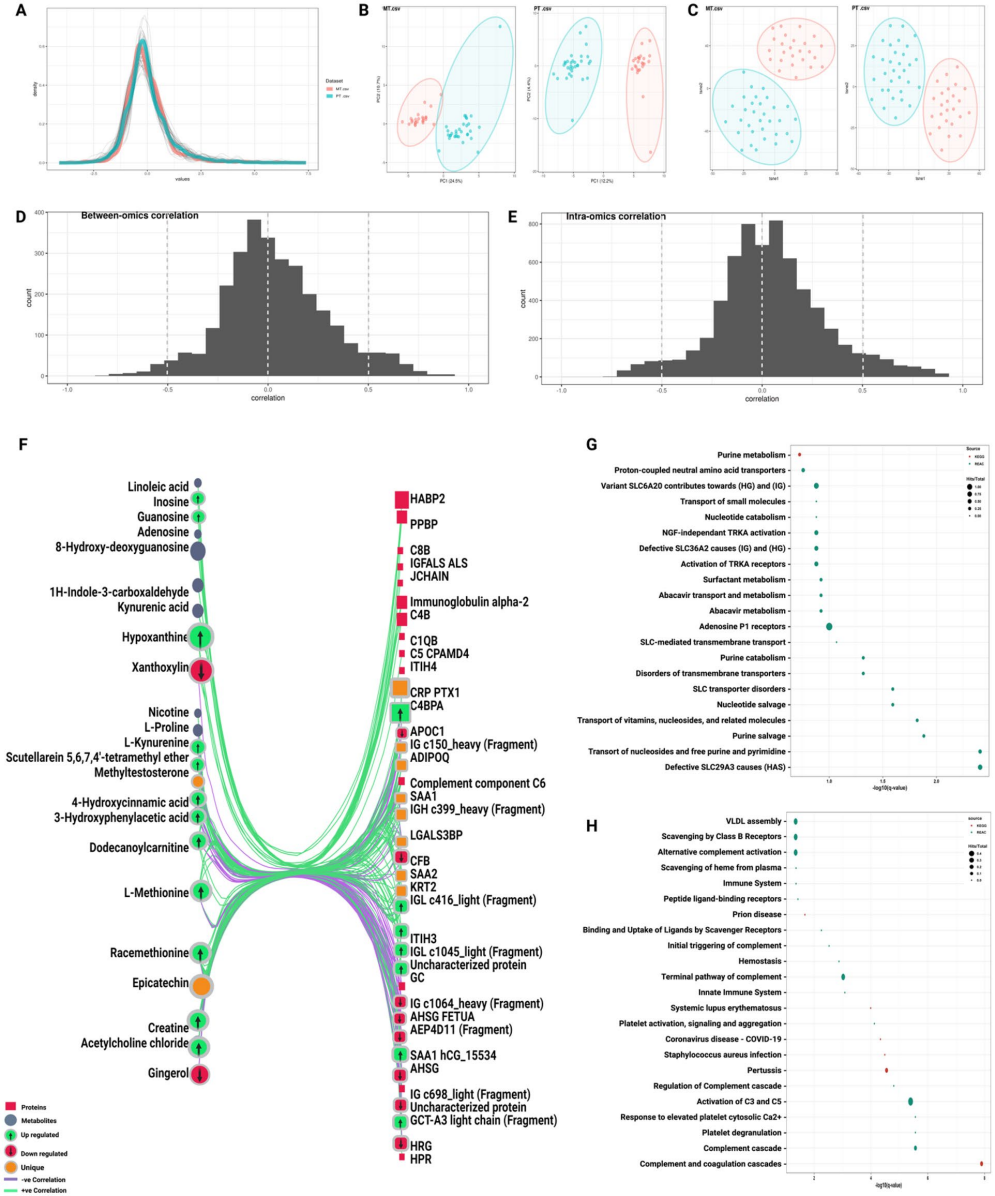
Statistical-based correlation between plasma proteins and metabolites. (**A**) Alignment and distribution of proteomic and metabolomic datasets after the auto-scaling and prior to integration. (**B**, C) PCA and t-SNE plots for both omics among healthy individuals and COVID-19 patients (**D**, **E**) correlation between and intra-omics datasets (**F**) The Statistical-based network contains 37 proteins labeled in red and 23 metabolites labeled in blue, with 64 negative correlation edges highlighted in purple and 179 positive correlation edges highlighted in green. (**G**, **H**) Pathway enrichment analyses applied on the statistical-based network and retrieved from KEGG (red) and Reactome (green) databases. The circle size reflects the number of identified hits in the pathway divided by the total entities within the same pathway. The x-axis represents the –log10 of the q-value.

The same set of proteins and metabolites used for the knowledge-based network was also used for constructing the statistical-based correlation network to pinpoint the possible significance between experimental plasma proteins and metabolites and to uncover the correlation and co-regulation pattern between biomolecules with total dependence on datasets of our profiles to avoid the bias towards the knowledge-based approach. After passing the above-mentioned data processing steps, both datasets were distributed within the same range on the density plot (Fig. 3A). Unfortunately, although PCA and t-SNE plots showed fair stratification patterns associated with the COVID-19 cohort in both proteins and metabolites datasets, the first 2 components explained less than 50% of the observations for both proteomics and metabolomics (Fig. 3B,C). The correlation network constructed based on the above-mentioned methodology for feature selection and correlation matrix construction resulted in only one continent subnetwork consisting of 60 nodes and 243 edges representing the statistical correlation between 37 proteins and 23 metabolites (Fig. 3F, Table S3). The overall positive and negative correlations were equally distributed between and intra-omics and among 243 correlation edges selected in the network, 179 edges were positive with correlation coefficient median of 0.6 showing a slight tendency toward the strong positive correlation. Meanwhile, only 64 edges were negative with correlation coefficient median of -0.55 showing a slight tendency toward the moderate negative correlation (Fig. 3D,E). The resulted network contained 19 upregulated features, 10 down-regulated features, and 10 unique features supporting the notion of high dependency on the quantitatively change proteins and metabolites than the knowledge-based approach. Pathways enrichment utilizing the correlation network entities has matched 71% of the single omics pathway signature by sharing 20 significantly enriched pathways (FDR < 0.05). Also, it shared 25 significantly enriched pathways with the knowledge-based network. Nevertheless, some differences in pathways related to plasma lipoproteins and fibrin clot formation were noticed (Fig. 3G,H). Sample spacing analyses were illustrated in 2D & 3D-scatter plots and showed no multi-dimensional overlapping between SARS-CoV-2 and control samples across the proteome and metabolome datasets (Fig. S5).

### Integrated knowledge-statistical network analysis

**Figure 4:**
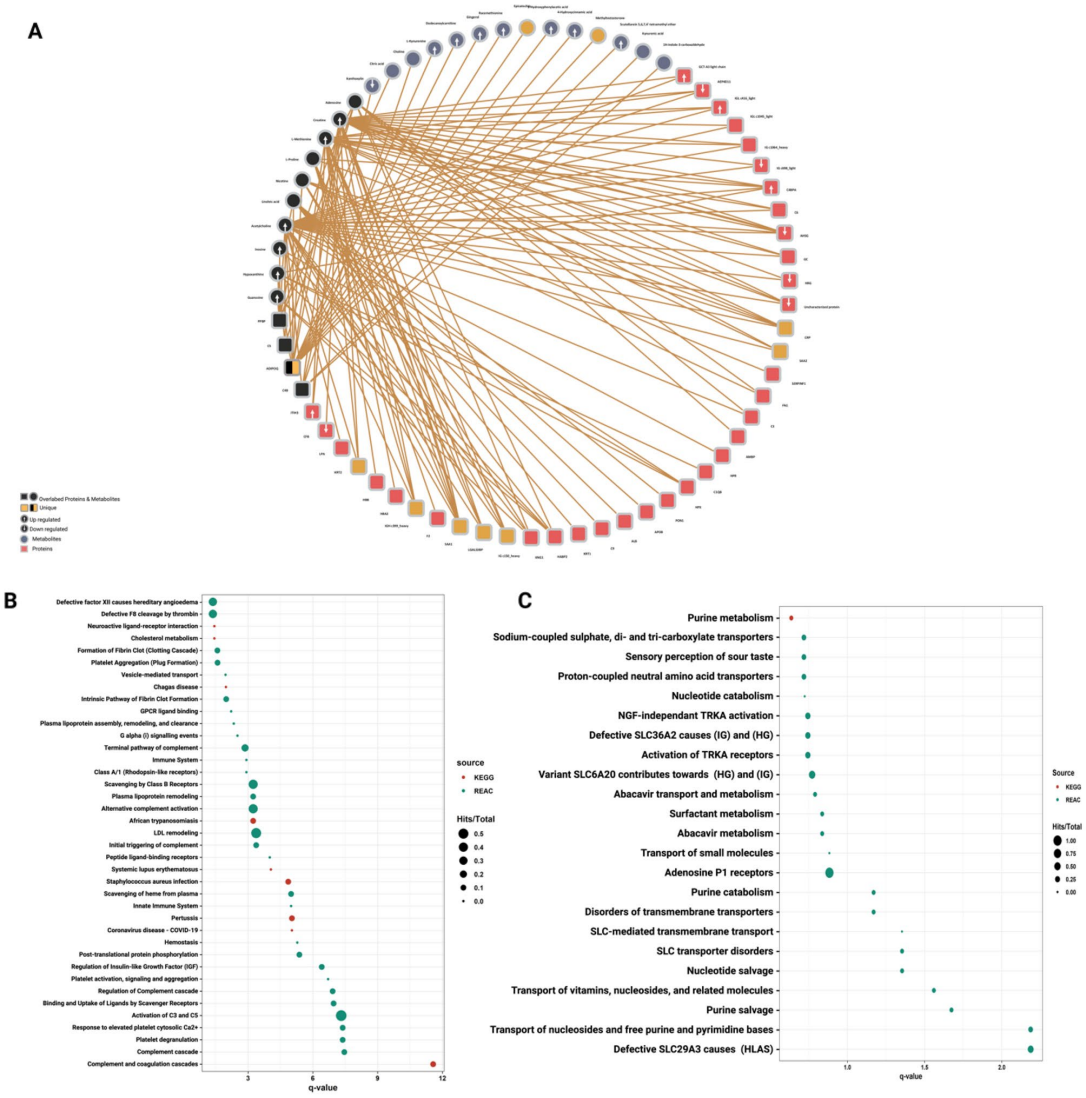
Integrated Knowledge-statistical-omics based network. (**A**) Integrated knowledge-statistical based network. The black colored nodes represent 14 overlapped features between the knowledge-based and the statistical-based networks interacting with 14 metabolites highlighted in blue and 39 proteins highlighted in red. Unique features highlighted in orange and each up or down regulated feature is marked with an up or down arrow as illustrated in the figure legend. (**B**, **C**) Pathway enrichment analyses applied on the knowledge-statistical based network and rerieved from KEGG (red) and Reactome (green) databases. The circle size reflects the number of identified hits in the pathway divided by the total entities within the same pathway. The x-axis represents the –log10 of the q-value.

The knowledge-statistical network fusion resulted in an integrative network built on 10 proteins and 4 metabolites found in both knowledge-based and statistical-based networks coupled with 53 features representing their first shell interactors from each network. The 14 shared features with the 53 interactors constructed the knowledge-statistical network with 67 nodes and 131 edges and contained 17 up-regulated features, 8 down-regulated features, and 9 unique features (Fig. 4A, Table S4). Significantly enriched proteomic and metabolic pathways by the knowledge statistical network had shared 79% with the single omics significant pathways. In addition to the overlapped pathways, the knowledge-statistical network uncovered a new set of pathways related to dysregulation of hemostasis like platelet aggregation (Plug Formation), defective factor XII causes hereditary angioedema and defective F8 cleavage by thrombin (Fig. 4B,C).

### Comparative network and pathway analysis among single and multi-omics approaches

**Figure 5:**
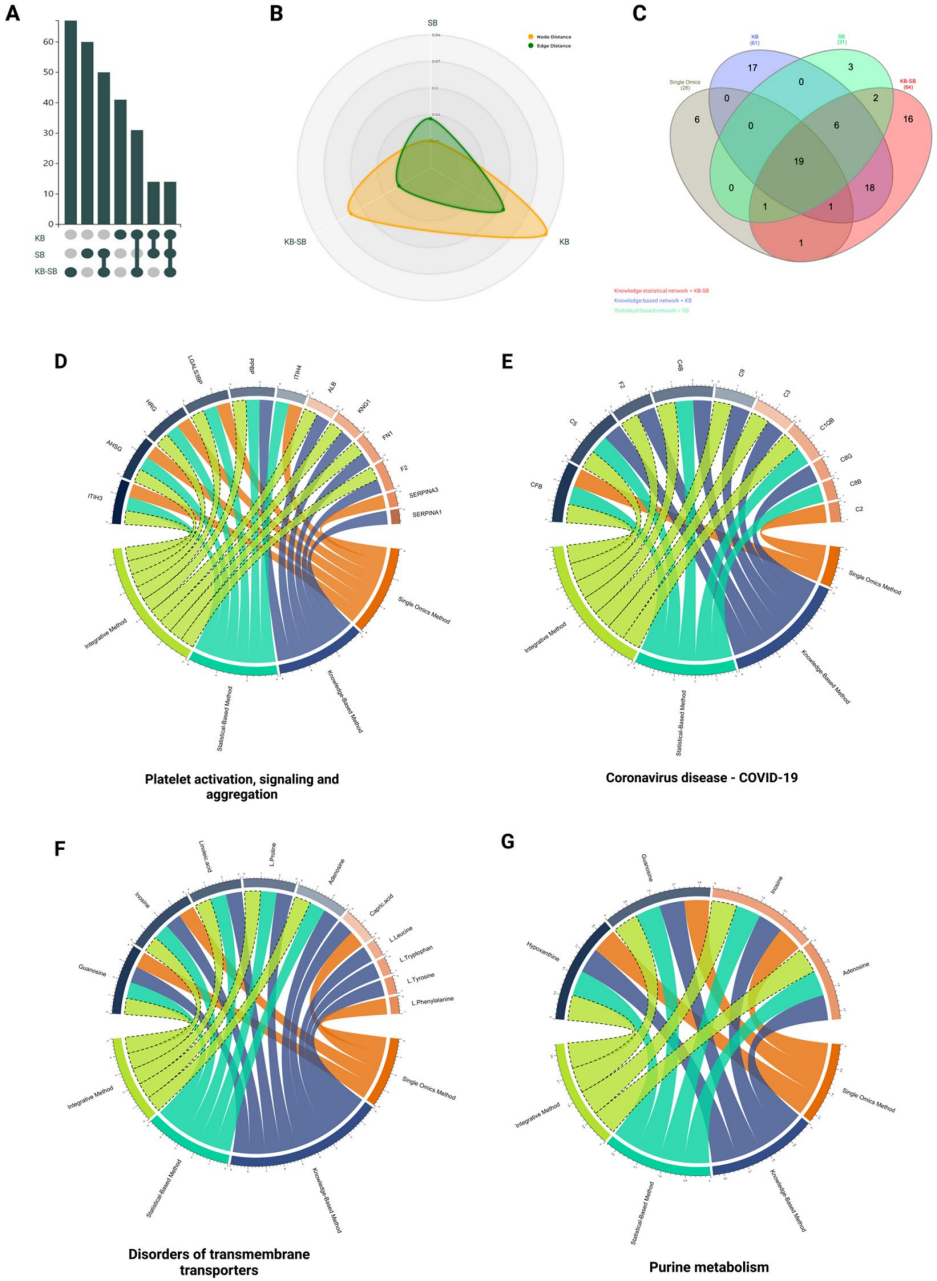
Networks comparative analysis and pathways tracking analysis. (**A**) UpSet plot comparing node size of each network generated from different omics method. The knowledge-statistical network (KB-SB) has the largest node size with 43 proteins and 24 metabolites. Meanwhile, the knowledge-based network (KB) has the smallest node size with 23 proteins and 18 metabolites and also revealed 14 overlapped features in the intersection between the knowledge-based and statistical-based networks. (**B**) Radar plot shows the similarity between the networks on both node and edge levels based on the distances between them, the node distance is highlighted in green and the edge distance is highlighted in yellow. (**C**) Significant pathways (FDR < 0.05) uncovered by each method were compared to each other and represented by the Venn diagram. (**D–G**) Four pathways were further tracked for a better understanding of how far each method can see inside the pathway, including (**D**) platelet activation, (**E**) coronavirus disease, (**F**) disorders of transmembrane transporters pathways, and (**G**) purine metabolism pathway. Each method was highlighted in a different color as illustrated in the chord plot.

The UpSet plot showed that the knowledge-statistical network has the largest node size and revealed 14 overlapped features in the intersection between the knowledge-based and statistical-based networks (Fig. 5A). The statistical-based network showed a shorter distance (higher similarity) toward the knowledge-statistical network on the Radar plot on both node and edge levels (Fig. 5B). This could be explained by the fact that the knowledge-statistical network depends on statistically correlated features more than the knowledge integrated features (more unleashed pathways). The Venn diagram showed that the knowledge-statistical network uncovered 64 significantly enriched pathways making it the highest approach, while the single omics approach uncovered only 28 significantly enriched pathways making it the least approach with the dimmest biological vision about the pathogen (Fig. 5C).

### Platelet activation, signalling and aggregation

Platelet activation, signalling and aggregation pathway is one of the chiefly affected pathways with the SARS-CoV-2 infection. It was enriched by the previously discussed methods, with differences in the key proteins involved in the pathway and the FDR values. The single omics method has 6 protein hits with 1 unique acute-phase protein called SERPINA3. Also, the knowledge-based method has 6 protein hits with 1 unique acute-phase proteinase inhibitor called SERPINA1. The statistical-based method also has 6 protein hits shared with the other methods.

Meanwhile, the integrative method outnumbered the other methods with 9 shared protein hits. Unsurprisingly, the integrative method has the most comprehensive biological insight due to its plethora of enriched proteins. The knowledge-statistical network did not introduce any unique hits in the pathway, which validates the method's integrity as it was initially built on the integration between the knowledge-based statistical-based networks. It is not supposed to introduce outsider hits (Fig. 5D).

### Coronavirus disease 2019 (COVID-19) pathway

COVID-19 pathway is a signature pathway for the SARS-CoV-2 infection describing all of the molecular interactions caused by the virus within the host cells. All omics analysis methods detected the COVID-19 pathway, but it was only enriched by the multi-omics approaches with 6 hits and 1.0e-4 FDR for the knowledge-based method, 5 hits and 4.62e-05 FDR for the statistical-based method, 7 hits and 9.31e-6 FDR for the integrated knowledge-statistical method. Therefore, more fidelity and confidence could be discerned from the integrated method.

The single omics method uncovered 2 hits in the pathway without passing the 0.05 FDR threshold. Interestingly, most of the protein hits in the COVID-19 pathway were involved in the complement cascade. The knowledge-based, statistical-based, and knowledge-statistical methods described the activation of the classical and lectin pathways, where they all end up forming a membrane attack complex that leads to cell death. In contrast, the single omics method detected only the complement component 2 (C2) at the beginning of the pathway, describing the initiation of the classical (Fig. 5E).

### Disorders of transmembrane transporters

Defects in SLC-mediated transmembrane transporters for amino acids, long-chain fatty acids, and nucleosides were highly noticed in SARS-CoV-2 patients. This defect causes respiratory complications, histiocytic, and renal disorders. Four metabolites, including L-Phenylalanine as a unique metabolite hit, were identified by a single omics method, and they were all enrolled in the defective SLC27A4, SLC29A3, and SLC6A19 pathways. Similarly, both statistical-based and knowledge-statistical methods uncovered the exact 5 metabolite hits that are involved in the same pathways. In contrast, the knowledge-based method uncovered 9 metabolites enrolled in the defective SLC27A4, SLC29A3, SLC6A19, and SLC36A2 pathways and uncovered L-Leucine, L-Tryptophan, and L-Tyrosine as unique metabolite hits (Fig. 5F).

### Purine metabolism

SARS-CoV-2 hijacks the host machinery to translate the viral mRNA and produce viral proteins, promoting viral survival and virulence^32^. This process intensively consumes the host nucleotide pool and forces the host cells to increase the purine nucleotides by upregulating the purine synthesis and, subsequently, dysregulating the purine metabolism^32,33^. Purine derivatives, including adenosines, guanosine, inosine, and hypoxanthine, have an essential role in regulating the pro-inflammation developed from the infection^34^. Three metabolite hits were reported in the single omics method, including guanosine, inosine, and hypoxanthine. In contrast, the other methods uncovered all the purine derivatives in the pathway (Fig. 5G).

## Discussion

**Figure 6:**
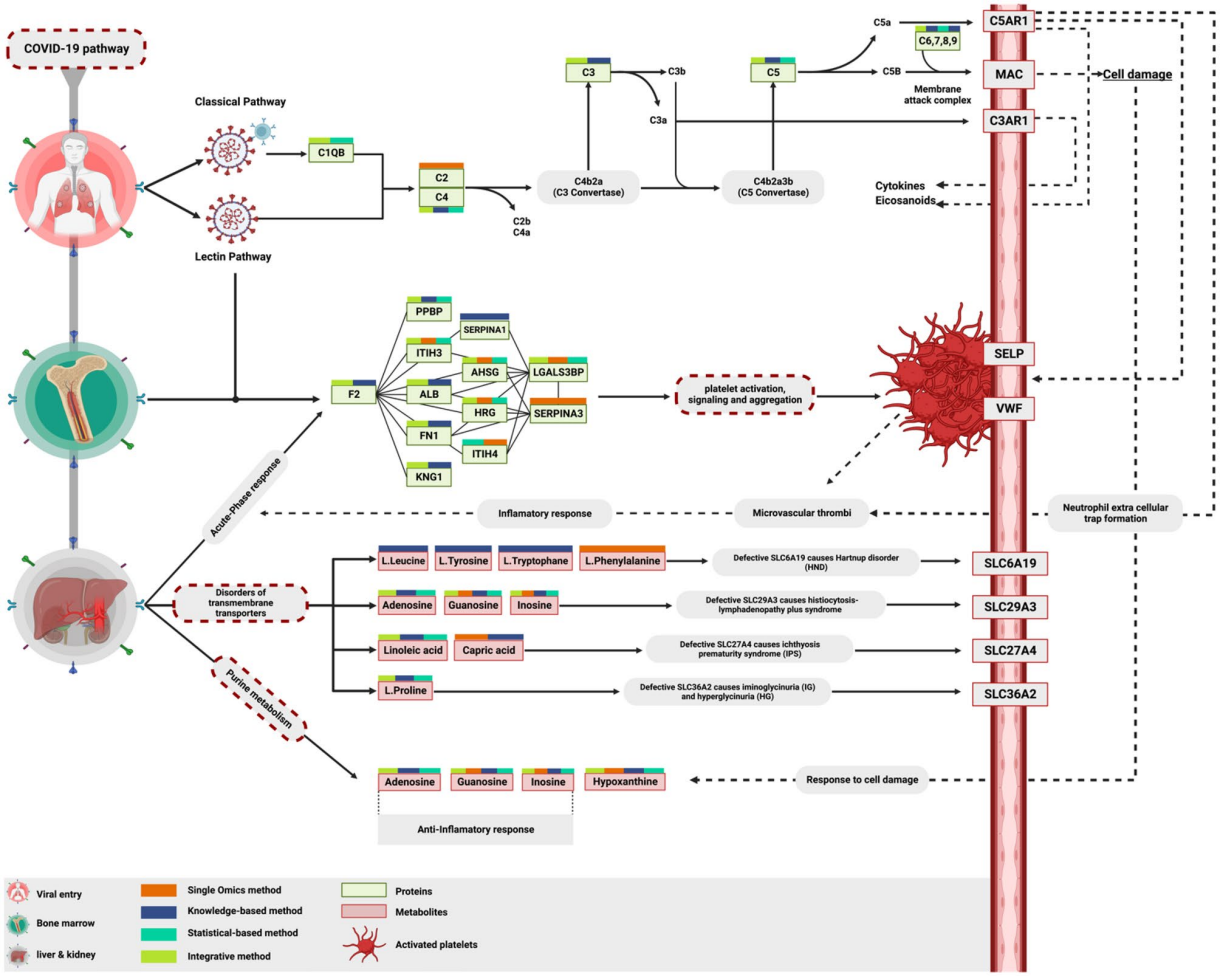
Key Proteins and Metabolites characterized by each omics method in severe COVID-19 Patients. Multi-omics methods were able to pinpoint the SARS-CoV-2 activation on both classical and lectin pathways with subsequent visualizing the whole complement cascade. This scenario leads to cell damage caused by the membrane attack complex, neutrophils recruited by chemoattractant like PPBP, inducing the elevation of various APPs such as F2, ITIH3, ITIH4, ALB, KNG, AHSG, and HRG. Additionally, it induces platelet activation, signalling, and aggregation. In contrast, the single omics analysis was able to uncover only one C2 in the initiation of the complement cascade and few APPs in the platelet activation pathway. On the metabolic side, the multi-omics approaches were able to uncover most of the hits in the “Disorder of transmembrane transporters” and “Purine metabolism” pathways compared to single omics.

To our knowledge, this is the first experimental study to compare the differences between single omics and multi-omics analysis in the COVID-19 research and to validate the impact of the multi-omics approaches over the conventional single omics analysis. We applied untargeted proteomics and metabolomics to profile plasma proteins and metabolites of a cohort of 32 Covid-19 patients with severe symptoms and analyzed their profiles to illustrate the molecular landscape uncovered by each analysis method. Multi-omics analysis was represented through three different networks: the knowledge-based network; which integrates the protein-metabolite interactions, the statistical-based network; which integrates statistically significant correlated proteins and metabolites; and the integrated knowledge-statistical network, which combines overlapped proteins and metabolites from the knowledge-based and the statistical-based networks with their first shell interactors from each network. Figure 6 illustrates the panoramic depth of each omics approach. The recruitment of single and multi-omics approaches depicted COVID-19 infection signature pathways at different levels. However, unambiguously, the peculiar ability of the multi-omics approach to unleash more pathway components was noticed (Fig. 6). For instance, the multi-omics approaches were able to detect the viral entry through both classical and lectin pathways, and the whole complement cascade started from the first component of the serum complement system (C1) and ended by forming membrane attack complexes (C6, 7, 8, and 9), causing cell lysis and death. This observation suggests that the complement system's suppression might benefit severe COVID-19 patients^35^. The single omics approach could pinpoint only complement component 2 (C2) at the cascade initiation. Various acute-phase proteins were released in response to the cell damage caused by the complement cascade. In contrast, multi-omics approaches uncovered most acute phase proteins in the platelet activation, signaling, and aggregation pathway and detected 6 unique functional proteins. Meanwhile, the single omics approach reported only 1 unique acute-phase protein. In response to tissue injuries caused by the robust immune response, pro-inflammation, clot formation, and cell damage, cells consume more ATP and produce more purine derivatives like adenosine, guanosine, and inosine, which have anti-inflammatory properties^33,36^. In this study, purine metabolism, signaling, and transport pathways were highly enriched by the multi-omics analysis. This finding confirms utilizing purine derivatives as a counterattack to orchestrate the immune regulation and anti-inflammation processes extracellularly through the purinergic signaling pathway^34,37,38^. Preferentially, the multi-omics approaches reported adenosine as a unique metabolite in the purine metabolism pathway.

On the other hand, the single omics approach shared three purine derivatives, including guanosine, inosine, and hypoxanthine, with the multi-omics approaches, which also carry an anti-inflammatory therapeutic function^34^.

Additionally, adenosine is a crucial immunomodulatory compound with the ability to convey immunoregulatory activity not only through interacting with A2AR & A2BR on the effector cells but also by recruiting other immunoregulatory mechanisms, including Tregs^39,40^. Added that adenosine is a crucial regulator that has the ability to out-compete the virus (SARS-CoV-2) for binding to the CD26 receptor^39,41^. Intriguingly, we found a wide range of transmembrane transporter disorders, especially amino acids, long-chain fatty acids, and most importantly, purine transporters which enhance purine levels extracellularly, leading to platelet activation and thrombosis reduction that may ameliorate inflammatory processes associated with coronavirus infection. The mechanism by which the cells make these transmembrane transporter disorders on purpose as an anti-virus attack is not clearly understood. However, it carries a lot of therapeutic potential and molecular insights for the pathogenesis of SARS-CoV-2 infection. For instance, if we could not control the transmembrane transporter selectivity toward a particular molecule, we could ultimately enhance the maintenance of the immunoregulatory and viral competitive molecules extracellularly.

Most severely diagnosed patients receive standard supportive care, antiviral and immunosuppressive therapies with minimal impact on COVID-19 patients that may even induce lung injuries. The uncovered molecular changes in this study might help to introduce new therapeutic strategies for severely diagnosed COVID-19 patients, mainly because the different multi-omics approach results' is not only aligned with several previously reported single proteomics or metabolomics articles that show molecular disturbance in central metabolic pathways like purine and amino acid metabolism but also have an extended vision and uncovers other molecular changes inside the same pathways^42–45^. In this study, both proteomic and metabolomic profiles were relatively quantified and required extensive intracohort validation for the most impactable proteins and metabolites. In addition, the impact of the drugs on the proteome and metabolome profiles should be evaluated (it has not been tested in this study). Further studies on plasma proteins and metabolites are required.

In conclusion, multi-omics employs different omics layers to provide deep molecular perspectives, analysis, and data interpretation. A shared similarities between each omics could be attained by either statistical inference or prior knowledge database retrieval. Our findings gleaned the unique, peculiar ability of multi-omics integrative knowledge-statistical- based approach over the single omics and other omics approaches in unveiling the molecular landscape of both inflammatory and anti-inflammatory processes for COVID-19. It is almost certain that the multi-omics integrative knowledge-statistical- based approach relies on both statistically correlated molecules and the prior knowledge-based cascade correlation. Gathering the benefits of both unleash new molecular interactions based on statistical correlation and relying on pre-defined knowledge-based cascade correlation. The versatile usage of this approach could provide a reliable mechanistic overview for better inferring versatile disease conditions.

## Data and code availability

Proteomics raw data files are available in PRIDE at https://www.ebi.ac.uk/pride/ under Project ID: PXD033335. Pride data are accessible using the reviewer email: reviewer_pxd033335@ebi.ac.uk and password: s0cnj4BY. Metabolomics raw files were publicly deposited on MassIVE at https://massive.ucsd.edu/ProteoSAFe/static/massive.jsp with dataset identifier: MSV000089568. Codes are available at https://gitlab.com/prolab11/Covid-19.

## Supplementary Information

Supplementary Information 1.

Supplementary Information 2.

Supplementary Information 3.

Supplementary Information 4.

Supplementary Information 5.
