# Supplementary material for: Integrated multiomics analysis to infer COVID-19 biological insights
Source: Sci Rep. 2023 Jan 31;13:1802. doi: 10.1038/s41598-023-28816-5 (PMC9888750; doi:10.1038/s41598-023-28816-5)
Supplement: Supplementary file 1 — Supplementary Information 1. [file 41598_2023_28816_MOESM1_ESM.docx]

**Supplementary Figures**

**Figure S1**

**
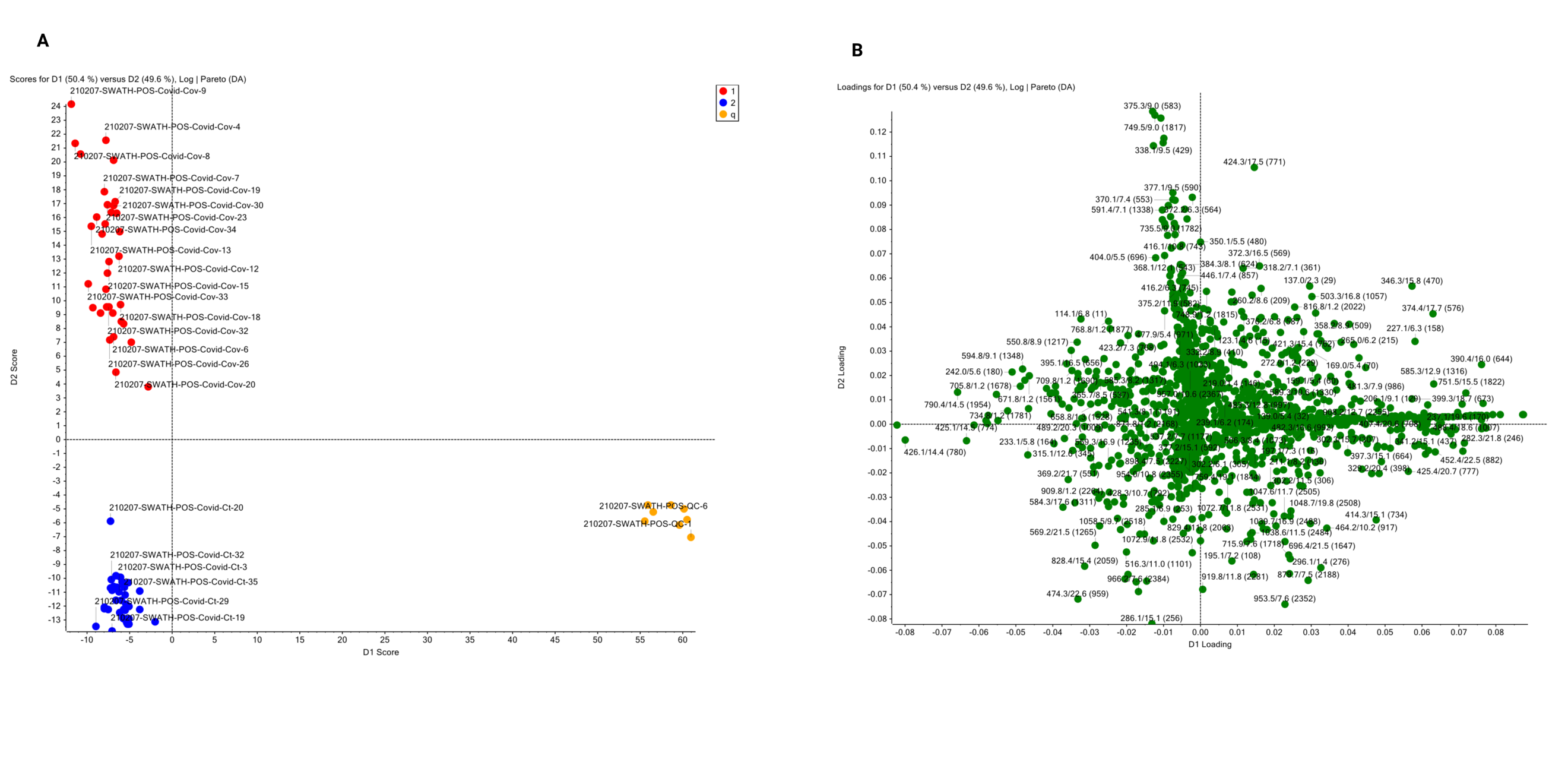
**

**Figure S1.** PCA analysis and loadings of control and Covid-19 cohort. Principle component analysis (left) and coordinates (right) showing the metabolite differences between control (blue) and Covid-19 patients (red). The PCA shows also the similarity of the pooled samples integrated within the runs to assure instrument stability.

**Figure S2**


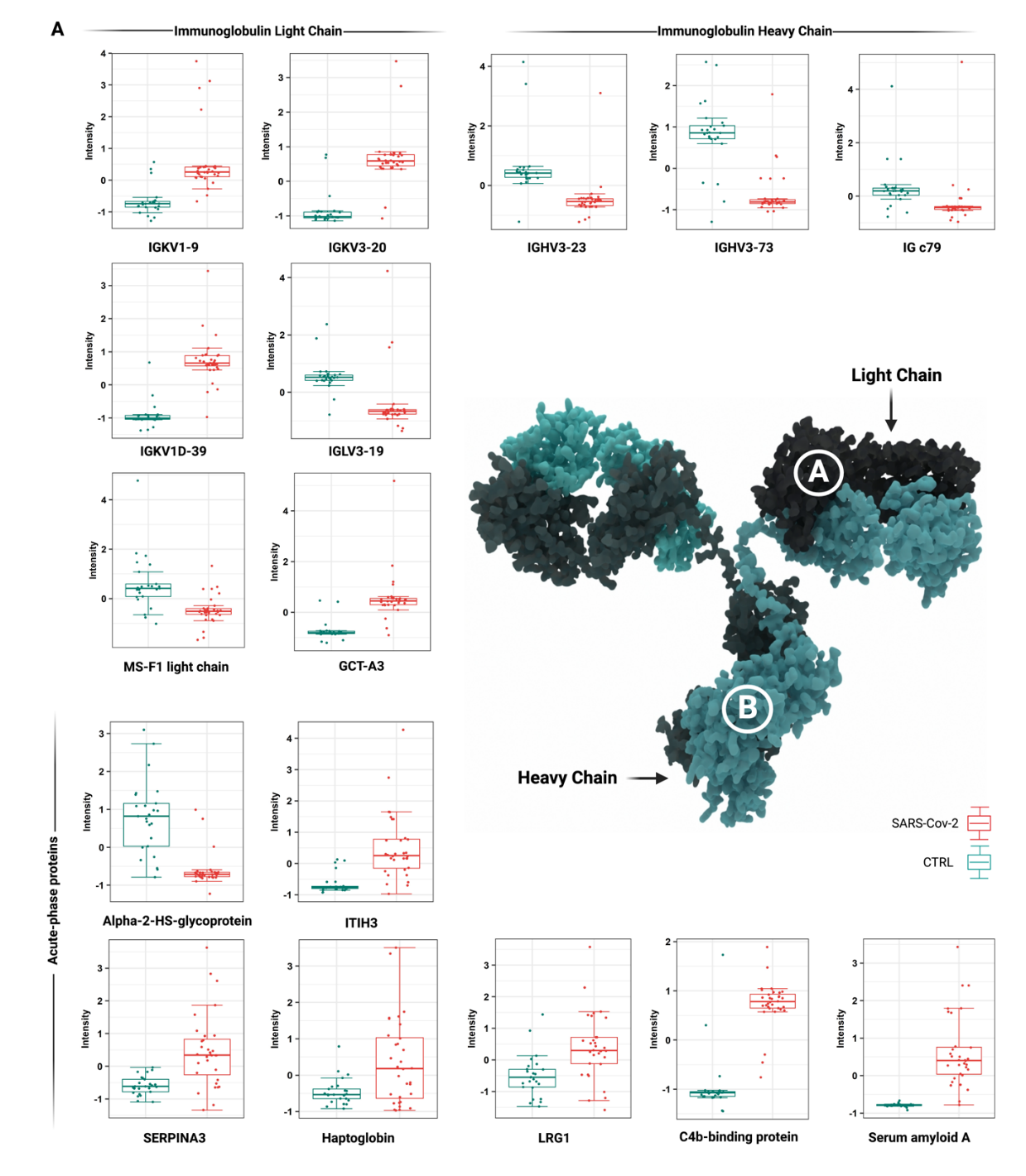


**Figure S2.** Box and whiskers showing the differentially expressed proteins between controls and Covid-19 patients. Significant proteomic changes caused by SARS-Cov2 infection are mainly affecting the immunoglobulins and acute-phase inflammatory proteins.

**Figure S3**

**
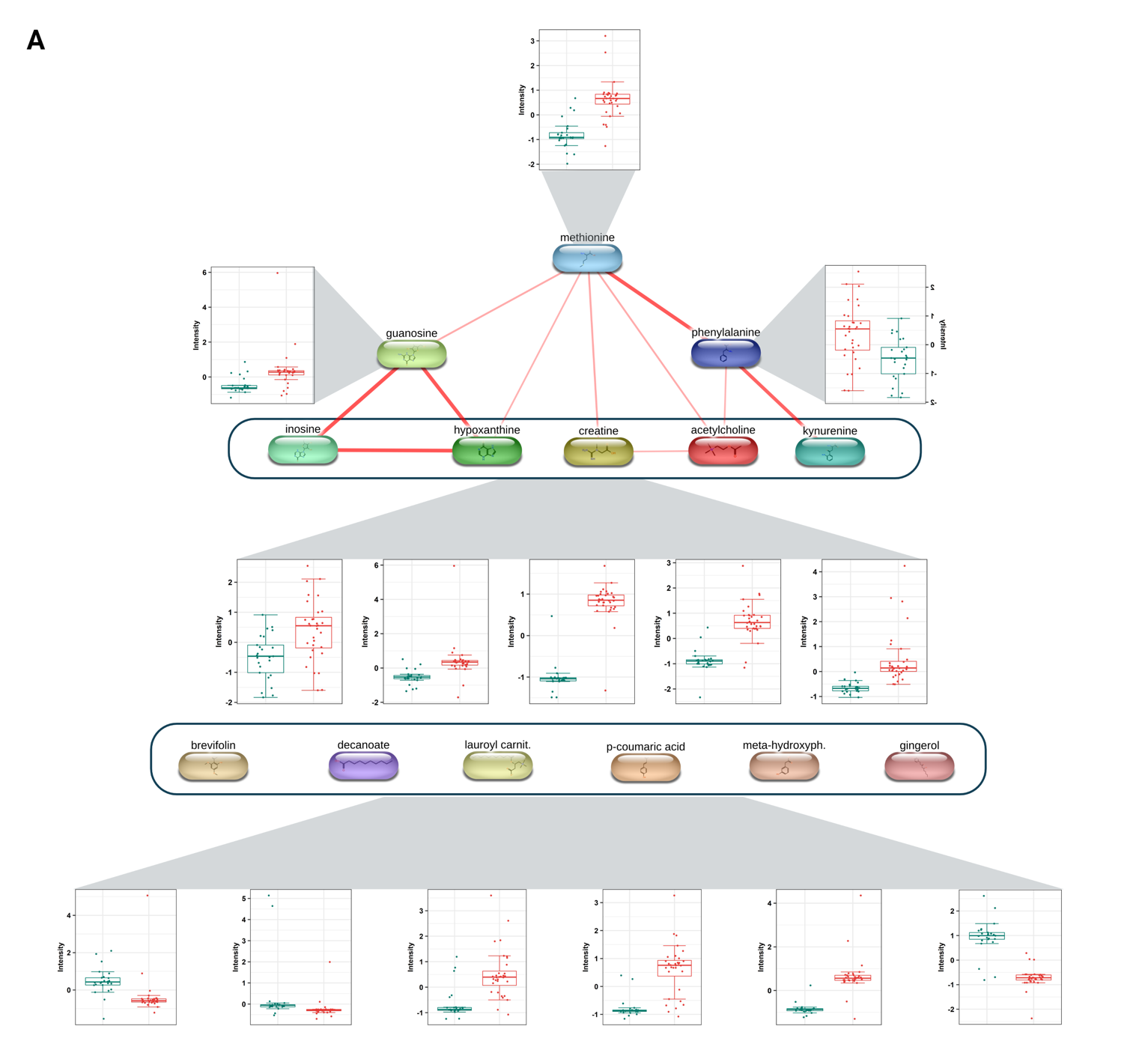
**

**Figure S3.** Differentially expressed metabolites between control and Covid-19 patients in single omics analysis. Significant metabolomic changes caused by SARS-Cov2 infection are mainly affecting the amino acids, purines and fatty acids derivatives.

**Figure S4**

**
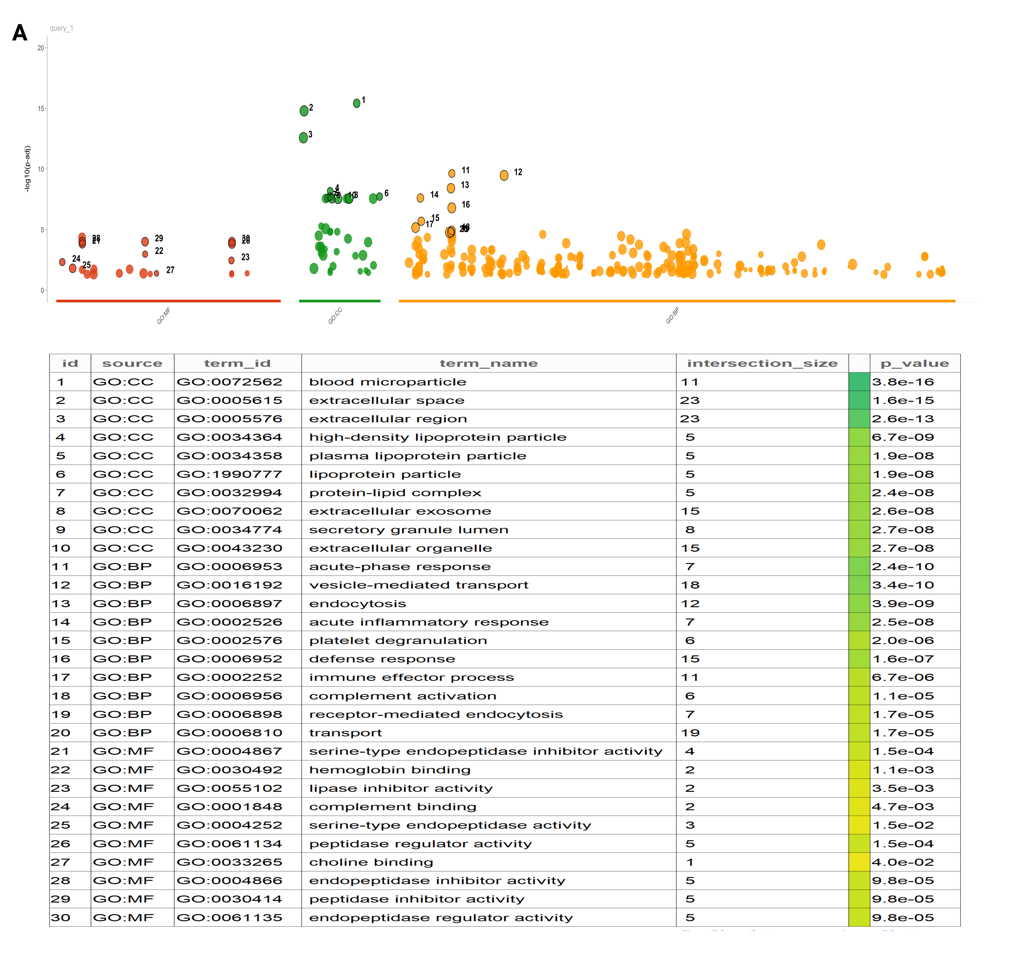
**

**Figure S4.** Gene ontology analysis of the untargeted proteomics identified significantly active acute phase and inflammatory biological processes with active extracellular secretory particles and lipase inhibitory activities.

**Figure S5**

**
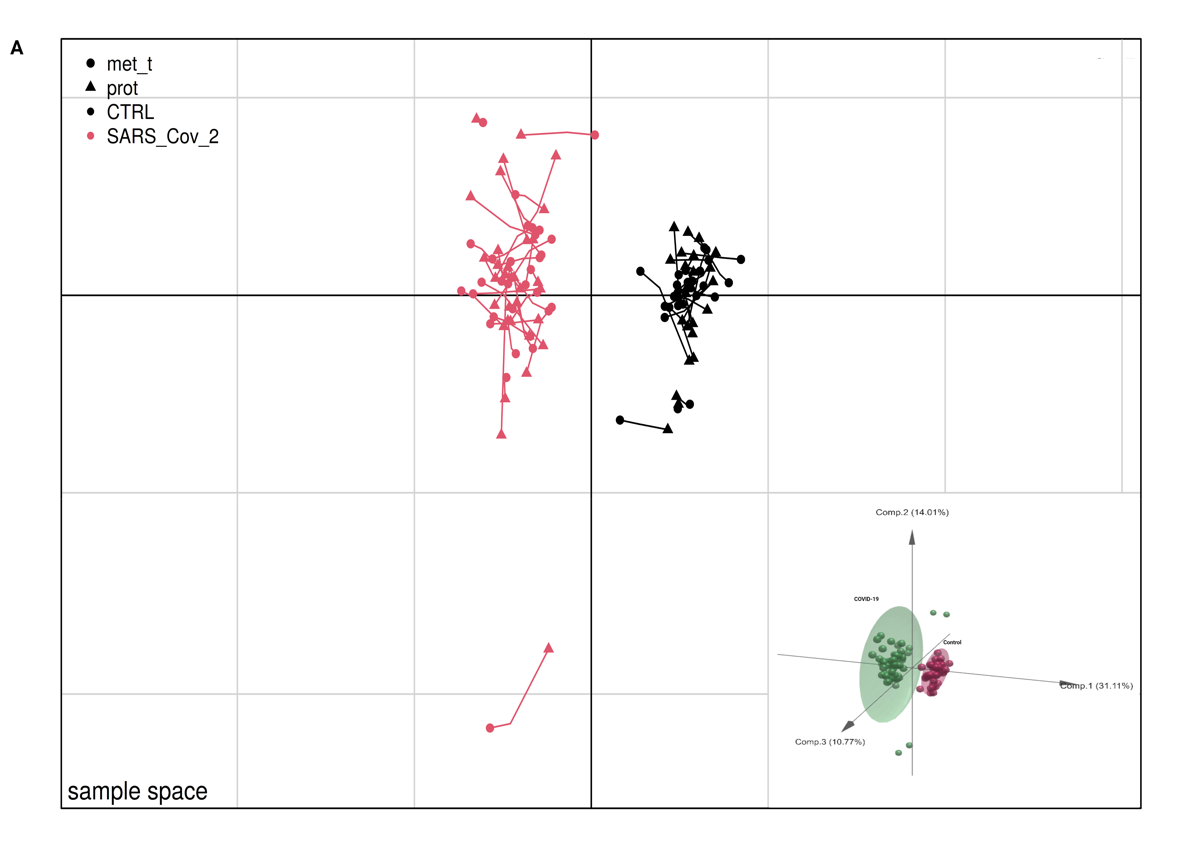
**

**Figure S5.** Multidimensional spacing between control and Covid-19 patients. Sample spacing analysis showed close individual correlation between patients same proteome and metabolome.
